# An Approach to Preschool Wheezing: To Label As Asthma?

**DOI:** 10.1097/WOX.0b013e3181fc7fa1

**Published:** 2010-11-15

**Authors:** Hugo P Van Bever, Eugene Han, Lynette Shek, Seo Yi Chng, Daniel Goh

**Affiliations:** 1 Department of Pediatrics, YLL School of Medicine, National University, Singapore

## Abstract

Asthma is considered a chronic disease, but not all preschool wheezing is asthma since most will eventually grow out of their symptoms. Although still a matter of debate, preschool wheezing can be classified in 2 major groups: virus-induced wheezing and multitrigger wheezing, having a different prognosis and a different treatment approach. Virus-induced wheezing is the most common phenotype of preschool wheezing and is usually associated with a good prognosis. Treatment should be conservative, but if preventive treatment is required, leukotriene-receptor antagonists might be the first choice treatment. Multitrigger wheezing is associated with an allergic disposition and has a higher risk of persistent symptoms. Inhaled corticosteroids may give short-term reduction in exacerbations, but the beneficial effect of long-term use of inhaled corticosteroids and other anti-inflammatory agents have not yet been established. This review aims to give an opinion on preschool wheezing, and its association with asthma.

## Introduction

Diagnosing asthma in preschool children is not always straightforward; it can be confusing or even impossible, especially when trying to demonstrate chronic airway inflammation. There is increasing awareness that recurrent preschool wheezing exists in several phenotypes that have different prognoses and management strategies. With its temporal nature, obvious relation to viral illnesses and lack of data on an underlying inflammatory process, recurrent preschool wheezing should not be synonymous with asthma.

From the few studies that used bronchial biopsies or bronchoalveolar lavage, it is clear that the inflammatory reactions in preschool children differ from those in older children with established asthma [1]. However, these studies were performed in those with severe wheezing or with unusual clinical features, which limits the value of the findings. The degrees of inflammation and compositions of the infiltrates were variable, with neutrophils dominating in some studies, eosinophils in others and no evidence of either in the rest [2].

The symptoms and signs of asthma in young children are commonly mimicked by a range of recurrent lower airway infections, such as bronchitis, bronchiolitis, and pneumonia. Thus, given the multifactorial nature of all wheezing disorders it is highly likely that a large number of clinical phenotypes exist, and that those phenotypes described in the literature are mainly the extremes of a broad spectrum of preschool wheezing disorders [3].

## Definition of asthma and preschool wheezing

Asthma is one of the most common chronic diseases of childhood, and is defined as a chronic inflammatory disease of the lower airways, leading to symptoms of recurrent wheezing and cough. Because a clear distinction between asthma and normality has never been established, many children may have clinical manifestations that are caught in the "gray zone." Examples of this are a child with 2 wheezing episodes within the last 3 months and a child with a prolonged cough for 2 weeks. Although both children cannot be labeled as normal *stricto sensu*, they also lack the full features of asthma.

Hence, asthma should be considered a syndrome, made up of different phenotypes and occurring at all ages, with symptoms that can start during infancy or childhood and can persist up to adulthood [4]. Although most definitions of asthma do not state the duration of the disease or number of exacerbations before the diagnosis is considered, it is generally accepted that the symptoms should last at least 6 months with at least 3 exacerbations experienced. A conscious effort must also be made to exclude other pulmonary diseases like bronchiectasis, structural airway disease and chronic lung disease.

The fact that most recurrent preschool wheezing does not show the chronicity that is expected in asthma indicates these children should not be considered the same entity. Instead, it makes more sense that the diagnosis of asthma is based on the prediction of the persistence of symptoms in these children. A recent task force from the European Respiratory Society (ERS) proposed a new classification of preschool wheezing that would guide management and prognostication [5]. The classification is certainly useful. However, not everybody agrees with the new classification and the discussion is still wide open, suggesting more research on the subject is needed.

## Diagnosing wheezing in young children

Wheezing is a symptom that is not always easily recognized. Other forms of noisy breathing, such as snoring or even any type of noisy breathing ("ruttles"), are often labeled as wheezing. Most parents describe wheezing as "any sound" or "difficult breathing" and there was less than 50% agreement between parents' and clinicians' reports of wheeze and asthma [6]. In another study, parents of 92 infants with noisy breathing were interviewed, beginning with an open questionnaire and then directed toward a more detailed description. Wheeze was the most commonly chosen word on initial questioning (59%). However, only 36% of them were still using this term at the end of the interview, representing a decrease of one third, whereas the use of the word ruttles doubled [7]. These studies reflect the degree of inaccuracy involved in the use of the term wheeze in clinical practice that may lead to over-diagnosis. This has potentially important implications in the assessment, management, and research in preschool wheezing.

## Phenotypes of preschool wheezing

Despite the inaccuracies in diagnosis, wheezing is generally accepted to be a common symptom in young children. In studies on Singaporean youngsters, it was found that 25% had wheezing before the age of 2 years [8]. Similar results were found in other studies, notably the Tucson Children's Respiratory Study where the prevalence of preschool wheezing in a cohort of 1,246 subjects was more than 30% [9]. In different population studies it was found that approximately 1 in 3 children has at least one episode of wheezing before their third birthday, and that the cumulative prevalence of wheeze is almost 50% at the age of 6 years [10]. Even with its high prevalence, there is still a lack of evidence regarding the pathophysiology and treatment of preschool wheezing. Preschool wheezing is a heterogeneous disease in which 2 major phenotypes can be distinguished: virus-induced wheezing and multitrigger wheezing.

***Virus-induced wheezing***, which accounts for around two-thirds of all preschool wheezing, is an intermittent form of recurrent airway obstruction with normal premorbid lung function and subjects are asymptomatic between episodes. As these children have a favorable prognosis, they only need supportive treatment. However, severe attacks induced by respiratory syncytial virus (RSV) and clinically diagnosed as bronchiolitis, can be associated subsequently with an increased risk of recurrent wheezing and allergic reactions during early childhood [11]. More recently, human metapneumovirus (HMPV) has been identified as a new respiratory virus that can induce wheezing and lower respiratory tract infections in otherwise healthy young children (mean age 11.6 months) [12,13]. Long-term effects of HMPV and its impact on severity and persistence of asthma need to be established. Furthermore, the role of rhinoviruses as a trigger of wheezing in early life, and as a predictor of asthma at the age of 6 years, became clear from recent studies, showing that rhinoviruses are the most common trigger of acute wheezing at all ages [14].

***Multitrigger wheezing ***is usually associated with allergy and is less prevalent in early life, manifesting during the school-going years. There is usually a family history of asthma and allergies. This form of wheezing tends to occur during and between episodes and is more likely to persist beyond early childhood, with associated significant deficits in lung growth up to 11 years of age [15].

The recent ERS task force proposed following after approach to preschool wheezing [5]:

1. For clinical purposes, preschool wheezing should be described in terms of its temporal pattern and classified as episodic (virus-induced) or multitrigger wheezing.

2. The use of the terms transient, late-onset and persistent wheeze should probably be limited to population-based cohort studies and should not be used clinically.

3. The term "asthma" should probably not be used in preschool children because data regarding underlying inflammation are lacking.

However, in real life, it can happen that a specific diagnosis (viral-induced vs multitrigger wheeze) cannot be made, and has to be delayed for a period of time until the clinical course of the recurrent wheezing becomes obvious (ie, transient vs persistent).

## Atopy and other risk factors

Atopy has been associated with persistence of asthmatic symptoms beyond preschool age and seems to be the major risk factor for an unfavorable prognosis. In the MAS study, a large cohort study performed in Germany, it was demonstrated that any allergic sensitization early in life increases significantly the risk of becoming asthmatic at an older age [16]. Therefore, it has been suggested that prevention of asthma should be focused on the prevention of allergic sensitization early in life.

At the moment, a lot of discussion is still ongoing regarding the role of early exposure to allergens and the subsequent risk of sensitization. For house dust mite and pollen, it is generally accepted that an increased exposure early in life increases the risk for subsequent sensitization and subsequent allergic disease. In contrast, a study from Manchester demonstrated that low house dust mite exposure early in life, increases the risk for subsequent sensitization to house dust mite at the age of 3 years [17]. The role of early exposure to pets is also controversial, as more and more data are showing that early pet exposure induces tolerance, instead of sensitization, especially in children from allergic families and especially in those exposed to dogs [18-20]. Besides allergy, other risk factors for the persistence of asthmatic symptoms beyond preschool age are male sex and severe RSV bronchiolitis [4].

## Diagnostic approach to preschool wheezing

History taking is a main diagnostic tool in preschool wheezing. The focus should be on confirming the presence of wheeze (as opposed to other types of noisy breathing), identifying its temporal pattern, assessing its severity, and identifying possible triggers.

Physical examination can be completely normal, or may reveal abnormal chest findings (hyperinflation, decreased air entry, wheeze). Signs of an underlying allergic constitution (rhinitis, conjunctivitis, eczema) may be present. It is also important to identify unusual or atypical clinical features that would suggest other underlying chronic pulmonary diseases.

Because there is an increased prevalence of allergy in preschool wheezing (around 30%), compared with healthy preschoolers (12%),[21] determination of an allergic disposition via skin prick testing is indicated in the assessment of preschool wheezing. This is especially so in those with underlying allergic constitution or family history of allergies. The Tucson group proposed an asthma index, based on family history and atopic features, to predict persistence of asthma [22]. By using that index, they were able to show that 95% of preschool wheezing with a negative index did not develop asthma between the ages of 6-13 years. In other studies, the presence of food allergy, especially to hen's egg, during the first 3 years of life was a risk factor for the persistence of asthma until school-going age.

Apart from skin prick testing, other investigations like viral isolation, radiologic imaging, pH probe studies (to detect gastroesophageal reflux) and measurement of fraction of exhaled nitric oxide have limited value in the work-up of preschool wheezing [5]. Spirometry is difficult to perform in young children, but impulse oscillometry is an emerging, noninvasive and accurate means of assessing lung function in children as young as 3 years of age [23].

The recent ERS task force recommends the following approach to the assessment of preschool wheezing [5]:

1. The pattern and triggers of wheeze, personal and family history of allergies, and household smoking should be assessed by history taking.

2. Wheezing reported by parents should be verified by a healthcare professional.

3. Allergy testing should be performed in children requiring long-term management.

4. Other investigations should be avoided unless the wheezing is unusually severe, therapy-resistant or accompanied by unusual clinical features.

## Pharmacological therapy

Apart from environmental control, allergen avoidance and parent education, pharmacological treatment should be focused on prevention of symptoms and on improving the long-term outcome of preschool wheezing.

### Acute Therapy

Acute symptoms are commonly treated with short-acting beta-agonists, systemic corticosteroids (prednisolone) and oxygen in severe cases. Oral prednisolone has not been shown to be useful in mild-moderate wheezing associated with viral infections [24]. The role of ipratropium bromide is less clear, although minimal additional effect to short-acting beta-agonists has been reported, especially in infants [25]. Theophylline is not longer recommended because of its potential side effects and its minimal bronchodilating effects [26]. Intravenous or inhaled magnesium sulfate has been insufficiently studied in preschool children, but from the available literature, it seems that intravenous magnesium sulfate is more effective than inhaled magnesium sulfate in older children with acute asthma [27].

### Preventive Therapy

#### Inhaled corticosteroids (ICS)

Most children with preschool wheezing will grow out of their symptoms and do *not *need any preventive treatment; especially when the symptoms are mild or infrequent. However, when symptoms are severe or frequent, the initiation of a preventive treatment will decrease symptoms and improve quality of life of the child and the family. Furthermore, by suppressing bronchial inflammation, the risk for basal membrane thickening, permanent bronchial damage and impaired lung growth may theoretically be reduced. However, it was never shown that preventive treatment with ICS is able to decrease the occurrence of persistent childhood or adult asthma [28].

The short-term effectiveness of ICS in young asthmatic children has been shown, including the reduction of symptoms, improvement of lung function, and suppression of bronchial hyperreactivity [29]. In general, it appears that ICS are more effective in multitrigger wheezing than in virus-induced wheezing, as a number of negative studies in virus-induced wheezing were published [30-32]. However, good comparative studies on the effect of ICS in different phenotypes of wheezing are still lacking.

However, recent studies were unable to demonstrate significant long-term effects or disease modifying effects of ICS in young asthmatic children. In a study on 285 young children with a high risk for persistent asthma, inhaled fluticasone (88 *μ*g twice daily) for 2 years was unable to change the development of asthma symptoms or lung function abnormalities during a third, treatment-free year. These findings do not support any disease-modifying effect of ICS after the treatment is discontinued [28]. This was also seen in older asthmatic children, where asthma symptoms recur when ICS is discontinued [33]. In another study on 411 wheezing infants, intermittent budesonide had no effect on the progression from episodic to persistent wheezing and no short-term benefit during episodes of wheezing in the first 3 years of life [34]. In yet another study on 129 preschool children with moderate-severe virus-induced wheezing, preemptive treatment with high-dose inhaled fluticasone reduced the use of rescue oral corticosteroids. However, this was at the price of decreased height and weight gain [35].

Taken together, it seems that ICS have a limited long-term effectiveness in young children with recurrent wheezing. Short-term effectiveness of ICS has been shown, but a clear disease-modifying effect was never demonstrated.

### Leukotriene-Receptor Antagonists (Montelukast)

Effectiveness of montelukast in preventing both virus-induced and multitrigger wheezing has been shown in a number of studies [5]. However, there are also studies that show no improvement, such as a recent multicenter study on the protective effect of montelukast on postbronchiolitis wheezing [36]. From comparative studies between montelukast and ICS published, it was concluded that the clinical effect of montelukast is comparable to that of a low dose ICS, and that montelukast might be more indicated to prevent virus-induced wheeze, while ICS should be used in cases of multitrigger wheeze [37,38].

A latest study in 2009 showed that preschool children with moderate-severe intermittent wheezing did not have increased episode-free days or decreased corticosteroid use with episodic use of either budesonide or montelukast in early respiratory infections. However, that study did show decreased severity of acute illness in children with positive asthma predictive indices [39].

### Long Acting Beta-Agonists (LABA)

Although it has been shown that LABAs have a bronchodilating effect in young children,[40] there are no studies on long-term efficacy or safety of LABAs, with or without inhaled corticosteroids, in young asthmatic children. Therefore, the usage of LABAs is not recommended in preschool children. In older children (6 to 17 years of age), a recent study of Lemanske et al was able to show that adding a LABA is the best way to go if asthma is uncontrolled with a low dose of ICS [41].

## Conclusion

If asthma is considered a chronic disease, then not all preschool wheezing is asthma because most will eventually grow out of their symptoms. Although still a matter of debate, it makes sense to classify preschool wheezing according to its long-term prognosis, as a distinction between virus-induced and multitrigger wheezing (ie, allergic wheezing) might better guide treatment and prognostication.

Virus-induced wheezing is the most common phenotype of preschool wheezing and is usually associated with a good prognosis. Treatment should be conservative, but if preventive treatment is required, leukotriene-receptor antagonists might be first choice. ICS also might be tried out in these kids. Multitrigger wheezing is associated with an allergic disposition and has a higher risk of persistent symptoms. It has been shown that ICS may give short-term reduction and prevention of exacerbations, but the beneficial effect of long-term use of ICS and other anti-inflammatory agents have not yet been established. Furthermore, a comparative study between the 2 wheezing groups on the effectiveness of ICS has never been published. Therefore, further studies should attempt to determine if continuous long-term administration of any preventive treatment is able to improve the long-term outcome of any type of preschool wheezing.

A practical approach could be the after:

*In a preschooler with recurrent wheezing, the possibility of an underlying allergy should be assessed (by skin prick testing or determination of specific IgE)*.

*If allergy is not present, the wheezing should be considered as nonallergic (ie, viral-induced wheezing), and usually little maintenance treatment is needed, as most of the children will grow out of it. Therefore, treatment should be focused on treating the symptoms (with beta-agonists, short coursed of prednisolone, etc). If the symptoms seem frequently a leukotriene-receptor antagonist might be considered in the first place, before starting ICS*.

*In contrast, if allergy is present (positive skin prick test or positive specific IgE) there is an increased risk that the child will continue wheezing beyond preschool age. In this case ICS might be necessary to control the underlying inflammation and to prevent symptoms. However, it has not been shown that ICS will prevent the progress toward childhood asthma beyond preschool age*.
